# Near-Infrared Photoluminescence Responses of Single-Walled Carbon Nanotubes Induced by Biomolecules Detected on a Microbead Surface

**DOI:** 10.1021/acsomega.4c07641

**Published:** 2024-10-23

**Authors:** Yoshiki Tachikawa, Masahiro Ito, Masaru Irita, Takunori Harada, Kazuo Umemura

**Affiliations:** † Department of Physics, Tokyo University of Science, 1-3 Kagurazaka, Shinjuku, Tokyo 1628601, Japan; ‡ Department of Medical Course, Teikyo Heisei University, 2-51-4 Higashi-ikebukuro, Toshima, Tokyo 1708445, Japan; § Research Institute for Science and Technology, Organization for Research Advancement, Tokyo University of Science, 1-3 Kagurazaka, Shinjuku, Tokyo 1628601, Japan; ∥ Department of Integrated Science and Technology, Faculty of Science and Technology, Oita University, 700 Dannoharu, Oita City 870-1192, Japan

## Abstract

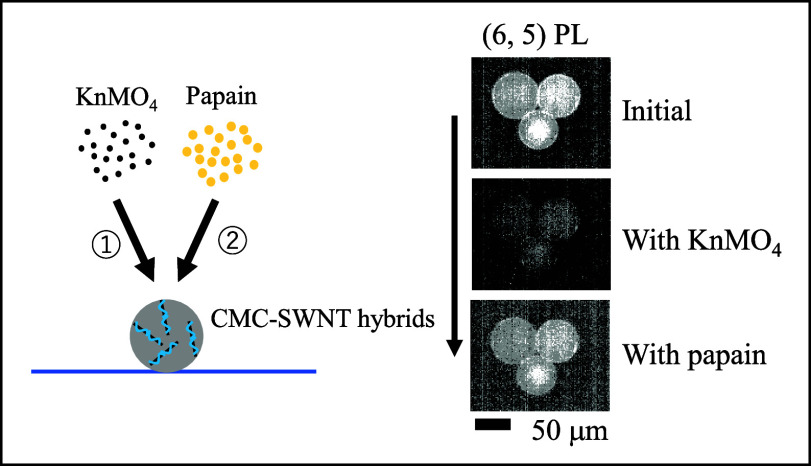

We demonstrate near-infrared (NIR) photoluminescence (PL) microscopy of single-walled carbon nanotubes (SWNTs) attached to individual micrometer-sized bead surfaces. Monodispersed SWNTs wrapped with sodium carboxymethyl cellulose (CMC-SWNT hybrids) were attached to the microbead surfaces, and the functionalized beads were immobilized on a coverslip. It is known that NIR PL spectra of the SWNTs were quenched upon exposure to potassium permanganate (KMnO_4_) and recovered by the addition of papain molecules by macroscopic PL spectroscopy. In this study, the PL responses were successfully detected on a microbead surface by NIR microscopy. In particular, PL responses of (6, 5) chirality SWNTs were selectively detected by using two bandpass filters. When KMnO_4_ (final concentration: 1 μM) was dropped onto the coverslip surface, the PL intensity of the SWNTs decreased to approximately 20% of the initial intensity without KMnO_4_. The average PL intensity of each bead was estimated from the microscopy images. When a papain solution (final concentration: 5 mg/mL) was added to the same sample, the PL intensity recovered to 60–70% of the initial intensity. A similar recovery was observed with papain solutions preheated at 60 and 100 °C. The microscopic observation of the beads was performed sequentially. Although the PL responses induced by papain molecules have been reported in previous studies using macroscopic PL spectroscopy, this is the first report to demonstrate responses on a single-bead surface. Our results hold significance for investigating enzyme reactions using SWNT PL responses at the nano and micro levels.

## Introduction

Single-walled carbon nanotubes (SWNTs) exhibit chirality-specific near-infrared (NIR) photoluminescence (PL) when excited by visible light.^1−4^ Chirality, which is described as a chirality vector (*m*, *n*), defines the structure and optical/electrical properties of SWNTs.^5−8^ For example, the excitation and emission wavelengths of (6, 5) SWNTs are typically 550–600 and 950–1000 nm, respectively.^9,10^ The PL wavelengths of SWNTs are shifted due to the environmental conditions; thus, the PL wavelengths of (6, 5) SWNTs vary among the previous reports.^11−13^ Even though there are wavelength shifts, chirality can be assigned by using PL spectra. The PL intensity of SWNTs also fluctuates significantly according to the conditions of the SWNTs.^14−16^ In general, the PL intensity of SWNTs increases or decreases when the SWNTs are oxidized or reduced, respectively.^16−18^ Although SWNTs exhibit strong PL intensity, their PL is quenched when bundled.^19^

The potential for biosensing based on the specific PL responses of SWNTs has been widely recognized by many researchers. Generally, monodispersed SWNT suspensions are prepared for biosensing approaches.^20−22^ DNA, surfactants, polymers, or other water-soluble molecules are mixed with SWNT powders, followed by sonication using homogenizers.^9,10^ Water-insoluble SWNT bundles are isolated by sonication, with water-soluble molecules wrapping the isolated SWNT surfaces.^23,24^ Hybrids of water-soluble SWNTs are useful for biosensing applications. In particular, SWNTs wrapped with DNA (DNA-SWNT hybrids) are frequently used because the hybrids are as stable as suspensions.^9,14,16^ For example, Polo and Kruss injected various biomolecules, such as dopamine and riboflavin, into polymer-wrapped SWNT suspensions to evaluate PL responses of SWNTs upon addition.^9^ Kurnosov et al. reported the effects of cysteine on SWNT PL.^16^ Those research studies have been mainly demonstrated with macroscopic PL spectroscopy using cuvettes with milliliter orders of SWNT suspensions.

Recently, microscopic techniques have been developed for the extensive observation of SWNTs. In particular, the PL of SWNTs injected into living cells has been intensively studied.^10,25−32^ For example, Kostarelos et al. directly observed cellular uptake induced by functionalized SWNTs. Sistemich et al. observed the production of dopamine in neural cells by SWNT PL.^10^ PL observations in vivo have also been reported recently.^33,34^ At the single SWNT level, Lee et al. succeeded in detecting the PL change in single SWNT hybrids caused by the injection of Ca using a homemade microscope.^35^ Roxbury et al. distinguished 17-chirality SWNTs by microscopic observation.^36^

The use of functionalized SWNTs with biomolecules, such as proteins, poses a technical problem. If the SWNTs form aggregates when biomolecules are added to the SWNT suspensions, PL measurements of the suspension become unstable.^37−39^ We found that DNA-SWNTs form large aggregates with papain molecules.^40^ We proposed a method to attach SWNTs to purified diatom frustules to solve the problem.^38^ Because diatom frustules are very light due to their nanoporous structures, they can be used as floatable microdevices.^41^ Using this method, the PL responses of the SWNTs induced by the injection of papain solutions were stably detected by macroscopic PL spectroscopy. The antioxidant properties of papain molecules were evaluated based on the PL responses of the SWNTs. However, the results were obtained from macroscopic PL spectroscopy and not from microscopic observations.

In this study, we demonstrated the microscopic observation of the PL responses of (6, 5) SWNTs on individual microbead surfaces. Two bandpass filters were used to detect specific PL emissions from the (6, 5)-chiral SWNTs.

(6, 5)-Rich SWNTs dispersed in sodium carboxymethyl cellulose (CMC) were attached to the microbead surfaces.^42−44^ Then, the functionalized beads were attached to a glass coverslip. As a test case, we observed the PL responses of the SWNTs induced by the addition of potassium permanganate (KMnO_4_) and papain molecules at the single-bead level by NIR microscopy. KMnO_4_ is a typical oxidant reagent; thus, it is known that SWNT PL is quenched by the addition of KMnO_4_ by macroscopic spectroscopy.^42^ We previously reported the detection of SWNT PL recovery by the addition of papain solutions by macroscopic spectroscopy.^37^ The PL recovery with papain was modest in contrast to that with the usual reductant chemicals, such as dithiothreitol. If PL responses can be detected on a single-bead surface, this will be the basis for establishing micrometer-sized NIR biosensors. In addition, thermal stability of papain molecules was evaluated by preheating papain solutions because papain is a typical thermostable protein.^45,46^ The previous research reported that papain molecules have enzyme activity even at 80 °C.

## Methods

(6, 5)-Rich SWNTs (773735; Sigma-Aldrich, Inc. St. Louis, MO, USA), CMC (1110, Daicel Miraizu Ltd., Osaka, Japan), papain (164-00172, FUJIFILM Wako Pure Chemical Co., Osaka, Japan), and glass beads (no. 0.05, 37 63 μm in diameter, Toshin Riko Co., Ltd., Tokyo, Japan) were used as received.

CMC powder was dissolved in a 10 mM Tris–HCl buffer solution (pH 8.0). The CMC solution and SWNT powder were mixed (weight ratio (CMC/SWNT) = 2:1; final concentration (CMC: 1.0 mg/mL and SWNT: 0.5 mg/mL)), followed by sonication (amplitude: 60%, frequency: 20 kHz, and power: 130 W) using a probe-type sonicator (VCX130, Sonics & Materials, Inc., Newtown, CT) for 90 min on ice. Next, 70% of the supernatant was centrifuged at 15,000 rpm (20,128*g*) for 180 min. The absorbance at 808 nm was used to determine the concentration of the suspension of CMC and SWNT hybrids (CMC-SWNT hybrids).^47,48^ The absorbance at 808 nm was suitable to define SWNT concentrations because it was not significantly affected by redox reactions. The absorbance of the suspension used in the following experiments was 1.34 when the stock suspension was diluted 3.7 times with the buffer solution. The prepared CMC-SWNT suspension was characterized by Raman spectroscopy (RAMANtouch 11i VIS-NIR, Nanophoton Corp., Osaka, Japan). The excitation wavelength used for Raman spectroscopy was 532 nm.

Fifteen milligrams of microbeads was suspended in 800 μL of dimethyl sulfoxide, followed by sonication in a bath-type sonicator (LEO-80, 46 kHz, 80 W, Tokyo Garasu Kikai Co., Ltd., Tokyo, Japan) for 30 min. 50 μL of 3-aminopropyltriethoxysilane (APTES; LS-3150, Shin-Etsu Chemical Co., Ltd., Tokyo, Japan), 12.5 μL of water, and 1 mL of acetic acid were added to the bead suspension.^49,50^ After incubation for 72 h at room temperature under gentle rotation, the suspension was centrifuged at 7000 rpm (3287 × *g*) for 5 min, and 70% of the supernatant was replaced with pure water. The solvent was replaced five times.

Next, 12.5 mg of 1-ethyl-3-(3-(dimethylamino)propyl)carbodiimide hydrochloride (WSC; 346-03632, FUJIFILM Wako Pure Chemical Co., Osaka, Japan) and 7.5 mg of *N*-hydroxysuccinimide (NHS, 081-09771, FUJIFILM Wako Pure Chemical Co., Osaka, Japan) were added to the aminated microbead suspension.^51,52^ Subsequently, 5 μL of the CMC-SWNT suspension was added to the microbead suspension and incubated for 48 h at room temperature under gentle rotation. The suspension was centrifuged at 7000 rpm (3287 × *g*) for 5 min, and 70% of the supernatant was replaced with pure water. The solvent was replaced five times. Microbeads containing the CMC-SWNT hybrids (CMC-SWNT beads) were stored as suspensions.

APTES aqueous solution (10%) was dropped onto a coverslip (2-176-12; Matsunami Glass Inc., Ltd., Osaka, Japan). After incubation for 1 h at room temperature in a sealed Petri dish, the coverslips were rinsed with water and ethanol and dried. 38.4 mg of WSC and 57.5 mg of NHS were dissolved in 1300 μL of *N*,*N*-dimethylformamide (DMF). Thirty microliters of the prepared solution was dropped on an aminated coverslip and then incubated for 1 h at room temperature in a sealed Petri dish. The coverslips were rinsed with water and ethanol and then dried. Thirty microliters of the CMC-SWNT beads was deposited on a functionalized coverslip and incubated for 24 h at room temperature in a sealed Petri dish. The coverslips with the immobilized CMC-SWNT beads were rinsed with water and ethanol and dried.

An inverted optical microscope (IX73; Olympus Co. Ltd., Tokyo, Japan) was used as the base. Two cameras, AdvanCam-E3RGc (AdvanVision Co., Ltd., Tokyo, Japan) and Xeva-USB-FPA-320 = 100 Hz (Xenics, Leuven, Belgium), were attached independently for visible and NIR observations, respectively. Halogen (100 W) and mercury lamps (100 W) were used as light sources for visible and NIR observations, respectively. For NIR observations, two single-band bandpass filters, FF03-575/25-25 (562.5–587.5 nm) (Semrock Inc., Rochester, NY) and FF01-1055/70-25 (1020–1090 nm) (Semrock Inc., Rochester, NY), were used for the excitation side and emission detection, respectively. The NIR observations demonstrated a high gain. The exposure period was 50 ms, and 50 objective lenses were used. The visible and NIR images were 1920 × 1200 pixel tif and 320 × 256 png files, respectively.

For visible and NIR observation, 500 μL of 10 mM phosphate-buffered saline (PBS, pH 7.0) was dropped on the coverslip with immobilized CMC-SWNT beads. The sample was first observed in the visible wavelength range, and then the same observation area was observed using the NIR setup (initial conditions). Subsequently, 1 μL of a 0.5 mM KMnO_4_ PBS solution was dropped on the coverslip for oxidation. The solution on the sample surface was mixed using a pipet. The same area was observed for each sample after incubation for 10 min at room temperature.

For standard evaluation of the effects of papain addition, the papain solution (10 mg/mL, PBS) was preheated at 60 and 100 °C for 1 h using a block incubator. Unheated papain solution served as a control. After the above NIR observation with KMnO_4_, 250 μL of PBS was sucked up from the coverslip surface. Then, 250 μL of the papain solution was dropped onto the sample surface. The solution on the sample surface was mixed using a pipet. After 10 min of incubation at room temperature, NIR images were recorded. The addition of PBS and preheated PBS at 100 °C without papain was also examined for comparison.

Circular dichroism (CD) and absorption spectra of papain samples (1 mg/mL) were measured using a CD spectrophotometer (JASCO: J-1500) in a Tris–HCl buffer solution at room temperature. The spectra were recorded over a wavelength range of 300–190 nm with “standard” sensitivity at 50 nm/min with 1 nm resolution and a time constant of 4 s. Data were further processed for noise reduction if necessary. The CD and absorption signals were presented as ellipticities (mdeg) and optical density. Percentages of the protein secondary structure motifs were estimated with CDPro software, containing the SELCON3, CDSSTR, and CONTIN/LL programs developed by Woody et al. We adopted the analysis of the program that provided the best fit among the three programs.

In the experiments involving the continuous addition of the papain solutions preheated at 60 °C, 28, 41, 78, 182, and 494 μL of the papain solution were sequentially added to the same sample after the addition of KMnO_4_. The final concentrations of papain on the coverslip surfaces were 0, 1, 2, 3, 4, and 5 mg/mL. NIR images were recorded after 10 min of incubation.

The averaged PL intensity of each bead was analyzed using Adobe photoshop.

## Results and Discussion

**Figure 1:**
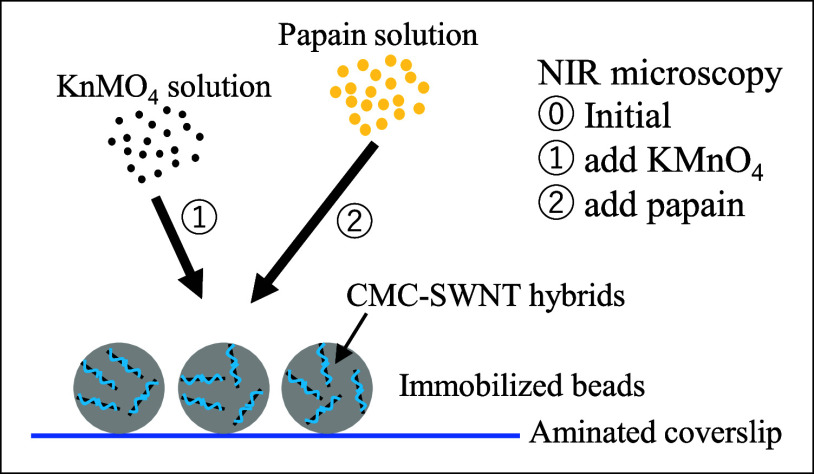
Schematic of the experiments. Micron-sized beads, decorated with CMC-SWNT hybrids, were immobilized on aminated coverslips. After NIR microscopic observation of the initial state, the NIR PL was quenched by the addition of KMnO_4_. Unheated or heated papain solutions were added to the same sample to detect PL recovery.

Figure 1 shows a schematic representation of this study. The CMC-SWNT hybrids were attached to micrometer-sized glass beads as antioxidant sensors. The functionalized beads were attached to glass coverslips using the established cross-linking protocol.^51,52^ By this treatment, the beads did not detach from the coverslip when the glass surface was rinsed with a buffer solution. The SWNT PL on individual bead surfaces was directly observed by using an NIR PL microscope. The PL responses induced by the addition of the oxidant and papain are shown in detail in Figure S1.

**Figure 2:**
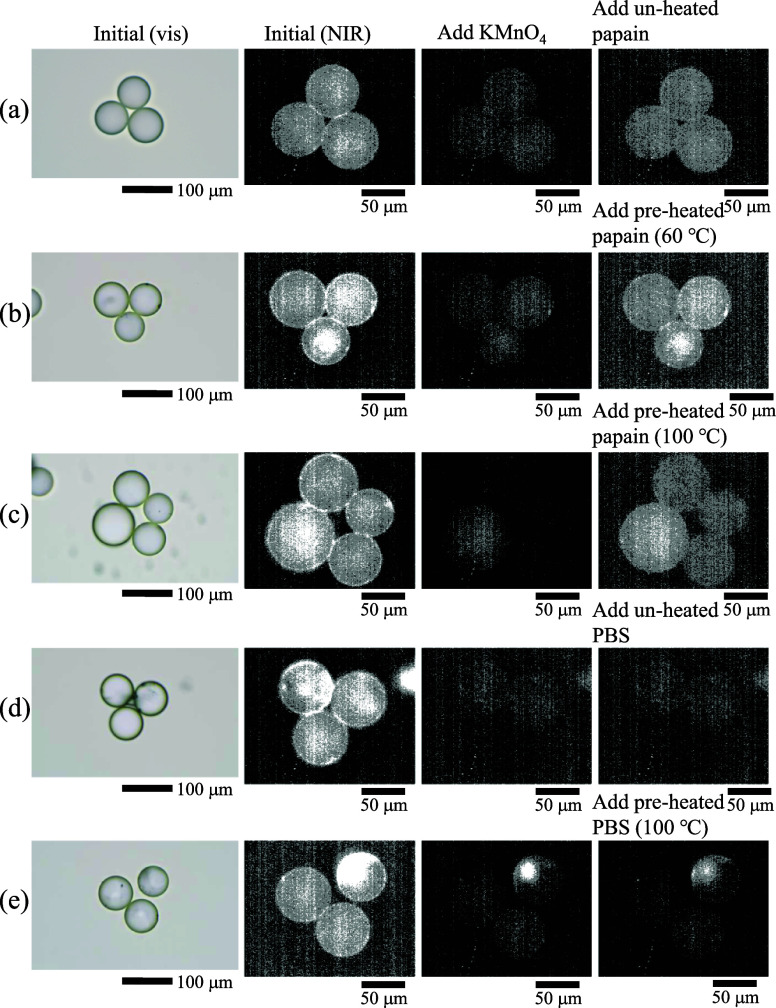
Visible and NIR microscopy images of functionalized micrometer-sized beads decorated with CMC-SWNT hybrids. Leftmost images: visible images of the initial samples. Second-left images: NIR PL images of the initial samples. Second-right images: NIR PL images after the addition of KMnO_4_ (final concentration: 1 μM). Rightmost images: NIR PL images after the further addition of papain solutions or PBS. (a) Addition of unheated papain. (b) Addition of preheated papain (60 °C). (c) Addition of preheated papain (100 °C). (d) Addition of unheated PBS. (e) Addition of heated PBS (100 °C).

Figure 2 shows the sequential microscopic images of the functionalized beads. Images obtained with visible light in the initial conditions revealed the typical circular shapes of the beads (leftmost images of conditions (a–e) in Figure 2). NIR imaging (excitation wavelengths: 562.5–587.5 nm and emission wavelengths (detection): 1020–1090 nm) also showed similar morphologies. The ranges of the excitation and emission wavelengths suggest that the emission originated from the (6, 5) chiral SWNTs, considering the shifts of the PL wavelengths caused by the CMC molecules.^53,54^ The beads were bright at the initial condition (second-left images of conditions (a–e) in Figure 2). These results correspond to the macroscopic PL spectra reported in previous studies using SWNT suspensions.^42^ The CMC-SWNT suspension before attachment to glass beads was evaluated by UV–visible absorbance spectroscopy (Figure S2) and macroscopic PL (Figure S3) and Raman spectroscopy (Figure S4). Typical (6, 5) SWNT emissions and standard SWNT Raman spectra were obtained. UV–visible absorbance data were used to estimate the concentration of SWNTs.^47,48^ Furthermore, modest peak shifts in the Raman spectra between the CMC-SWNT suspension and CMC-SWNT attached to microbeads were examined (Figure S5). The peak of the G-band shifted from 1596 to 1589 cm^–1^ after attachment to the microbead surfaces.^55,56^ The shift might be caused by stress due to the attachment to the solid surfaces.^53,57^

The oxidant, KMnO_4_ (final concentration: 1 μM), was then added to the samples. The emission from the SWNTs obviously disappeared (second-right images for conditions (a–e) in Figure 2). These results indicate that the NIR optical responses of the SWNTs, which have been measured by macroscopic PL spectroscopy with SWNT suspensions, could be observed on the bead surfaces. Finally, the unheated papain solution, preheated papain solution (60 °C), preheated papain solution (100 °C), PBS, and preheated PBS (100 °C) were added to the samples in conditions (a), (b), (c), (d), and (e). The purpose of this comparison was to evaluate the effects of preheated papain molecules on PL recovery of SWNTs. For the three papain samples, the SWNT emissions were modestly recovered from the far-right images under conditions (a), (b), and (c). The antioxidant abilities of papain molecules were observed as described in our previous study, which demonstrated macroscopic PL spectroscopy with functionalized diatom frustules decorated with DNA-SWNTs. When PBS was added to the samples, no PL recovery was observed (far-right images under conditions (d) and (e) in Figure 2).

**Figure 3:**
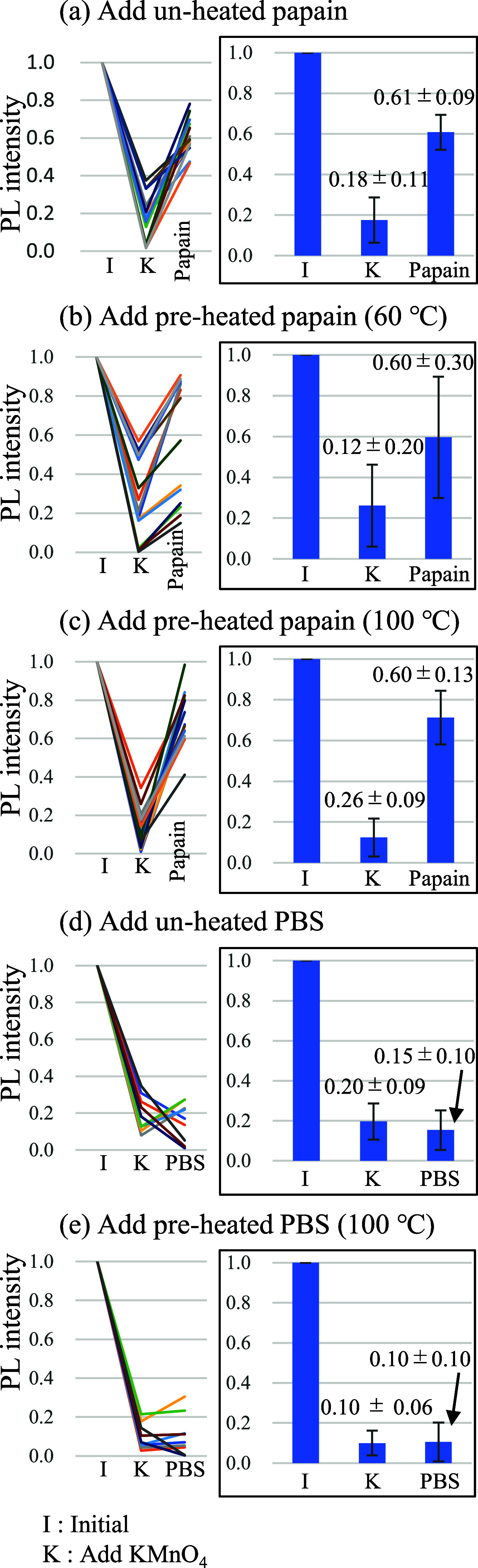
Sequential measurements of NIR PL intensities at the initial states after the addition of KMnO_4_ and after the further addition of papain/PBS. (a) Addition of unheated papain. (b) Addition of preheated papain (60 °C). (c) Addition of preheated papain (100 °C). (d) Addition of unheated PBS. (e) Addition of heated PBS (100 °C). PL intensities at the initial states were normalized as 1. *n* = 15 for parts (a), (b), and (c). *n* = 9 for (d) and (e).

Figure 3 shows the changes in PL intensities of individual beads due to the sequential addition of KMnO_4_ and papain/PBS. Intensity of one bead was determined by integrating the intensity of each pixel of the bead. The initial PL intensity of one bead was normalized to 1. The histograms show the average PL intensity of independent 15 beads (papain) or 9 beads (PBS) with standard deviation. Although fluctuations in the data were not negligible, quenching of PL by the addition of KMnO_4_ and recovery of PL by the further addition of papain were clearly observed (Figure 3a–c). The quenching with KMnO_4_ was demonstrated in order to effectively detect the modest PL recovery with biomolecules as did in previous macroscopic reports.^37,38^ Recovery was not detected with the further addition of PBS (Figure 3d,e). The numerical values of the data are summarized in Table S1.

The significance of PL recovery with papain was confirmed by using a *t*-test (Table S2). The *p*-values of (1), (2), and (3) are less than 0.05, whereas the *p*-values of (4) and (5) are greater than 0.05. We defined the null hypothesis as the absence of a difference between paired populations and evaluated it using a *t-*test. The null hypothesis was rejected for (1), (2), and (3); in these cases, there was a statistically significant difference.

To visualize the fluctuation in the measurements, the PL intensity of the individual beads was plotted in line graphs (left graphs of Figure 3). Data from 15 beads (papain) and 9 beads (PBS) were overwritten. The values of the beads are connected by a straight line. Further improvements are necessary to minimize these fluctuations.

PL recovery due to the addition of papain was 60–70%. Antioxidant abilities of papain molecules were not lost even after incubation for 1 h at 100 °C. This may indicate the thermostability of the papain molecules. Upon the addition of unheated PBS, the PL intensities were 20 and 15% of the initial PL intensities before and after the addition of PBS, respectively. Both were 10% of the initial PL intensity before and after the addition of preheated PBS. These results suggested that the PL responses that appeared in the papain solution were not due to the effects of the buffer solutions. The stability of pH during the observation was confirmed by using pH test papers.

Secondary structures of the unheated and heated papain molecules were evaluated using CD spectroscopy (Figure S6 and Table S3). As a result, helical structures of papain molecules were damaged by annealing at 100 °C. Further studies are necessary to understand the PL responses induced by the heated papain molecules.

**Figure 4:**
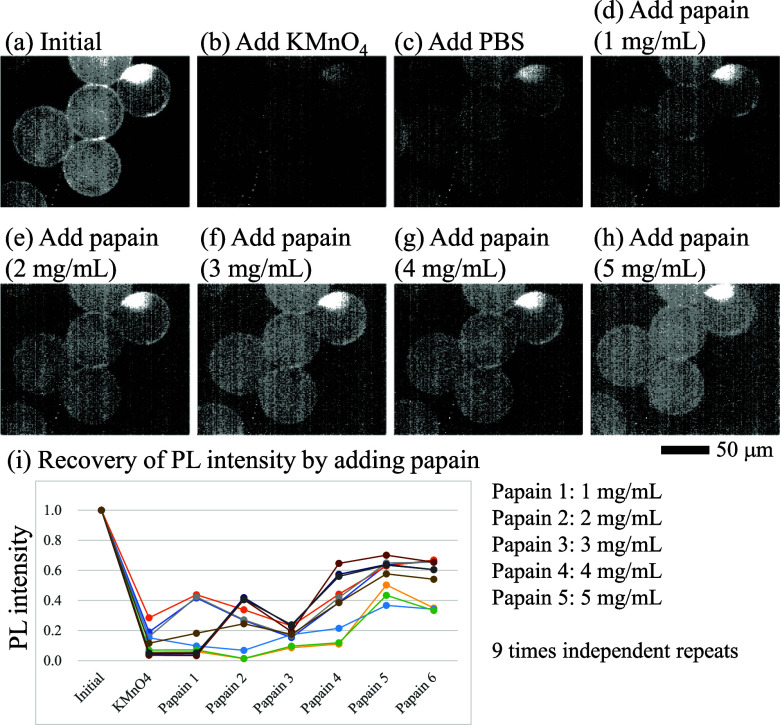
NIR microscopic observation of functionalized microbeads, decorated with CMC-SWNT hybrids, after the sequential addition of KMnO_4_, PBS, and preheated papain solutions (60 °C). (a) Initial state, (b) after addition of KMnO_4_ (final concentration 1 μM), (c) after addition of PBS (10 μL), (d) after addition of papain (final concentration: 1 mg/mL), (e) after addition of papain (final concentration: 2 mg/mL), (f) after addition of papain (final concentration: 3 mg/mL), (g) after addition of papain (final concentration: 4 mg/mL), and (h) after addition of papain (final concentration: 5 mg/mL). Interval of each observation was 10 min. (i) Sequence of the PL intensities. *n* = 9.

The continuous addition of a papain solution was demonstrated (Figure 4). After the reaction was quenched with KMnO_4_, the papain solution was gradually added to the same sample. Although these fluctuations were not negligible, the PL intensity gradually recovered with the continuous addition of papain. Sequential data from five independent beads are listed in Figure 4. The average values are plotted in Figure S7 with standard deviations.

Our results indicate that the NIR optical responses of SWNTs induced by the addition of protein molecules were well detected on individual micrometer-sized beads using an NIR microscope. Although the fluctuations in the obtained data are large, we believe that the establishment of a micrometer-sized optical sensor using NIR responses is valuable. In addition, a specific PL from the (6, 5) SWNT was successfully detected using a combination of bandpass filters, even with a mercury lamp. This indicates that the specific optical responses of SWNTs with various chiralities can be selectively used to study the physicochemical properties of biomolecules using various filters. In future studies, employing a laser instead of a mercury lamp is anticipated to yield a more stable and stronger optical response.

For sample preparation, the functionalized beads were immobilized on a coverslip. In this treatment, the same beads were continuously observed, even after the addition of an oxidant or a protein solution. Beads can also be used as a suspension. Because the CMC-SWNTs were attached to the microbead surfaces, aggregate formation with papain molecules was not fatal. In this case, the beads can be easily collected by centrifugation after use; thus, recycling the functionalized beads is possible.

## Conclusions

The NIR optical responses of the (6, 5) SWNTs induced by the addition of papain molecules were successfully detected on individual micrometer-sized bead surfaces. The responses from the (6, 5) SWNTs were specifically detected using bandpass filters. The new method will be applicable to the study of enzyme reactions using the NIR PL of SWNTs at the nano/micro level in future work.

## Supporting Information Available

Scheme of the sample preparation, visible absorbance spectra of CMC-SWNT suspension, PL spectra of CMC-SWNT suspension, Raman spectra of CMC-SWNT suspension, peak shift of Raman spectra of CMC-SWNT, numerical analysis of PL intensity, *t*-test of PL data, CD/absorbance spectra of papain molecules, secondary structures of papain molecules, and sequential measurements of PL intensity (PDF)
