# Supplementary material for: Near-Infrared Photoluminescence Responses of Single-Walled Carbon Nanotubes Induced by Biomolecules Detected on a Microbead Surface
Source: ACS Omega. 2024 Oct 23;9(44):44734–40. doi: 10.1021/acsomega.4c07641 (PMC11541476; doi:10.1021/acsomega.4c07641)
Supplement: Supplementary file 1 — ao4c07641_si_001.pdf [file ao4c07641_si_001.pdf]

Supplementary materials

Near-infrared photoluminescence responses of single-walled carbon nanotubes induced by biomolecules detected on a microbead surface

Yoshiki Tachikawa[1], Masahiro Ito[2], Masaru Irita[3], Takunori Harada[4], Kazuo Umemura[1*]

[1]Department of Physics, Tokyo University of Science, 1-3 Kagurazaka, Shinjuku, Tokyo 1628601, Japan
[2]Department of Medical Course, Teikyo Heisei University, 2-51-4 Higashi-ikebukuro, Toshima, Tokyo 1708445, Japan
[3]Research Institute for Science and Technology, Organization for Research Advancement, Tokyo University of Science, 1-3 Kagurazaka, Shinjuku, Tokyo 1628601, Japan
[4]Department of Integrated Science and Technology, Faculty of Science and Technology, Oita University, 700 Dannoharu, Oita City 870-1192, Japan

Corresponding author*
Kazuo Umemura
umemura@rs.tus.ac.jp

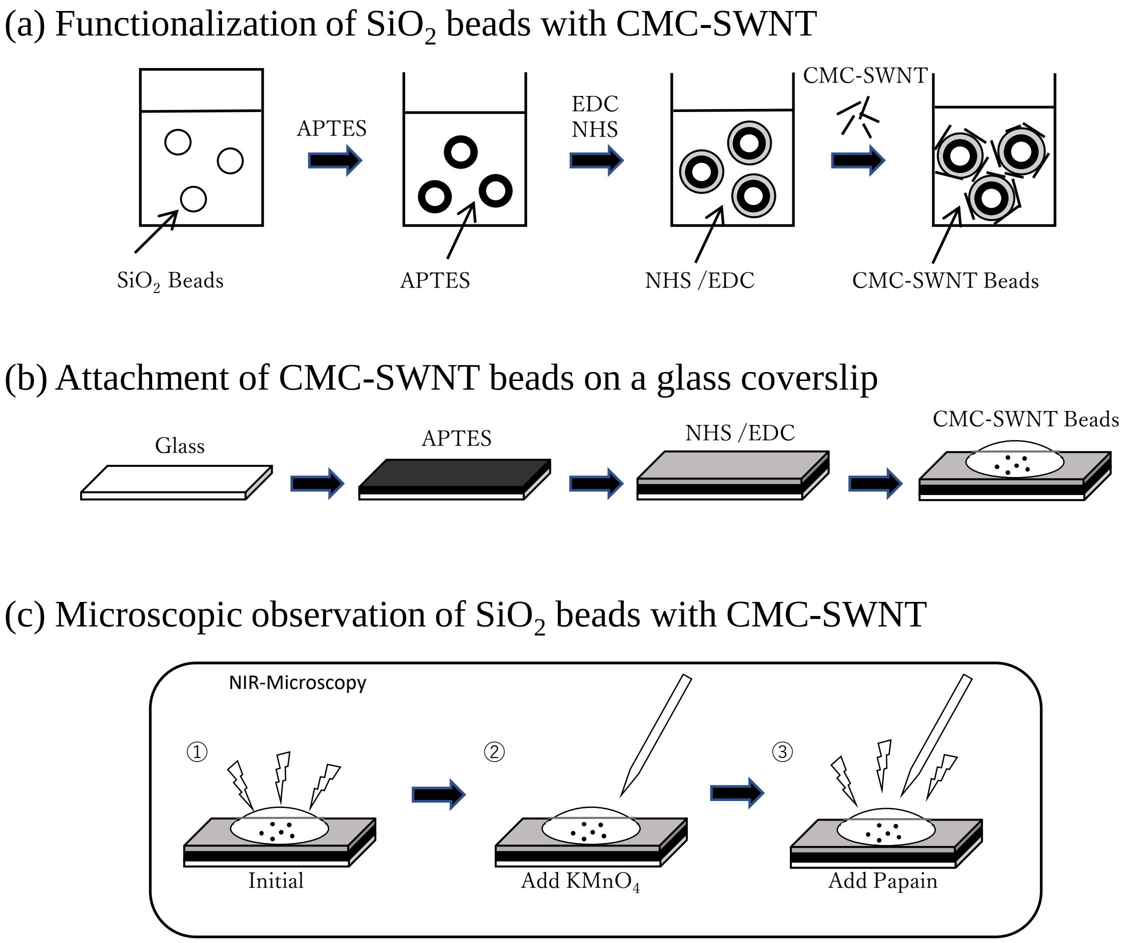

Figure S1
Scheme of the sample preparation.

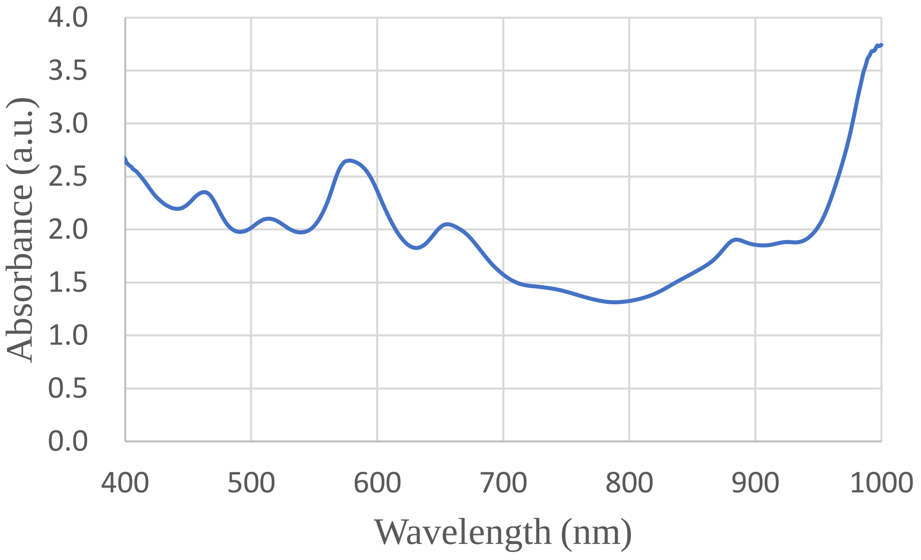

Figure S2
Visible absorbance spectra of the CMC-SWNT suspension.

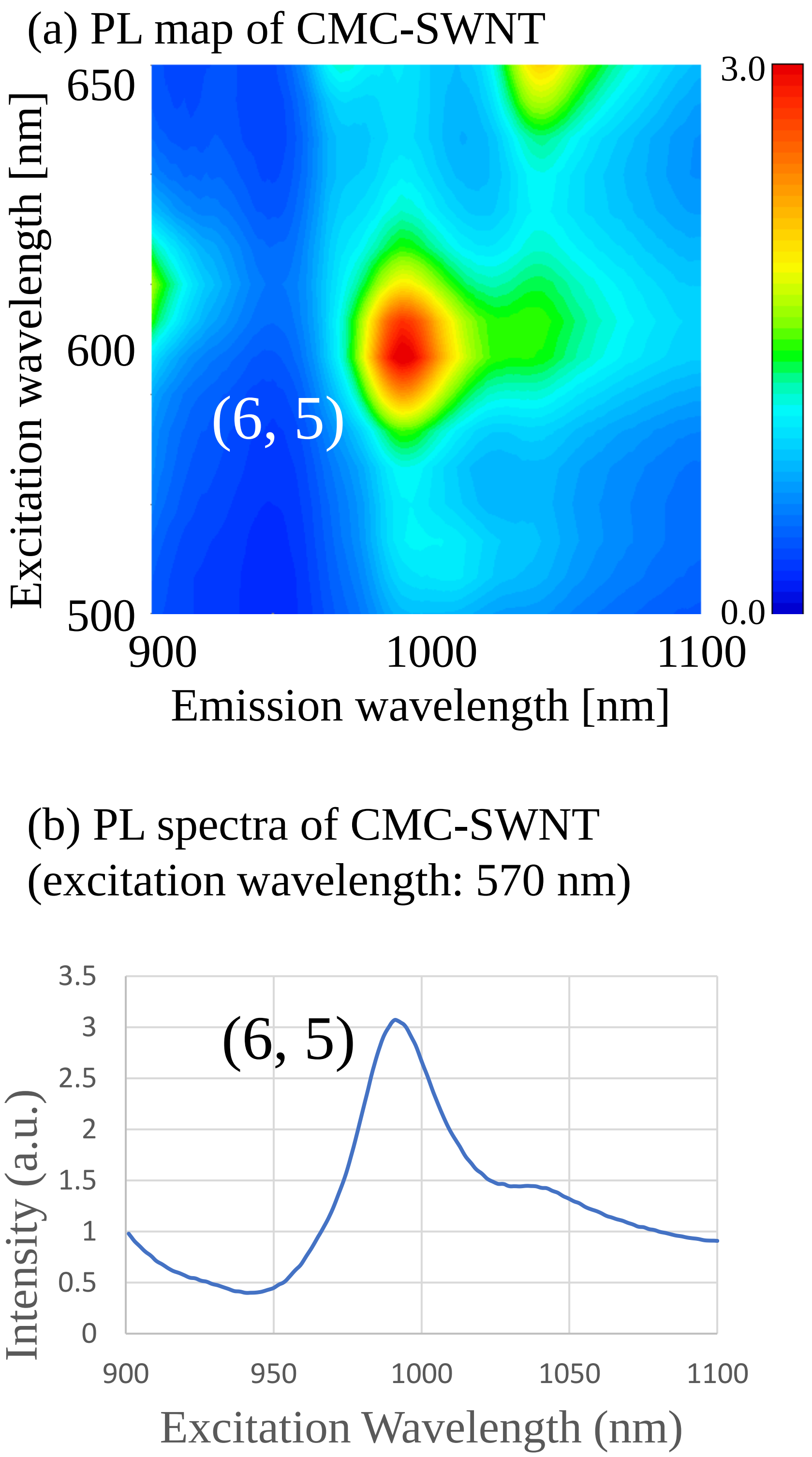

Figure S3

PL spectra of the CMC-SWNT suspension. (a) PL map. (b) PL spectra of (6,5) SWNTs. Cross-section of (a) at excitation wavelength 570 nm.

(a) Raman spectra of CMC-SWNT

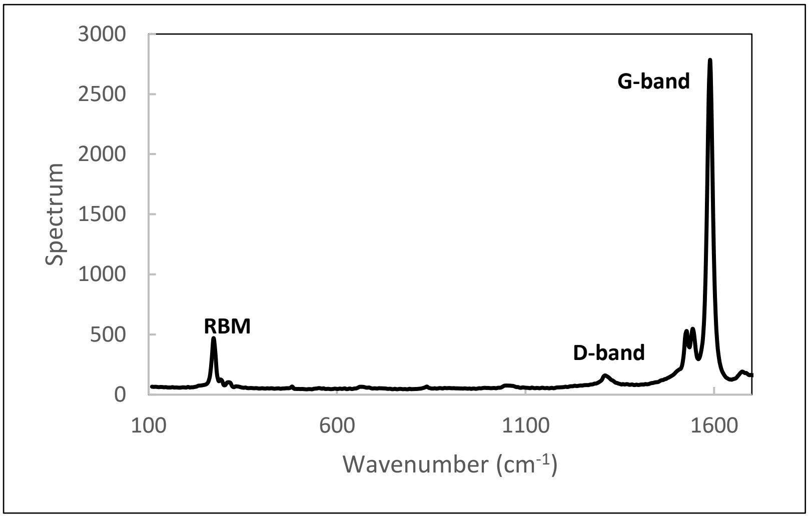

(b) Raman spectra of CMC-SWNT (RBM)

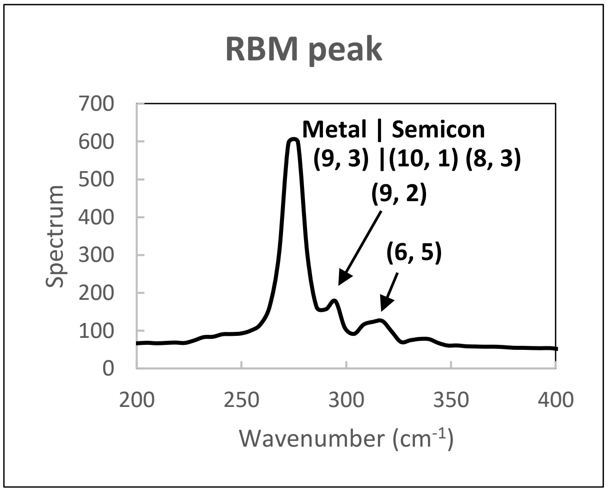

Figure S4

Raman spectra of CMC-SWNT suspension: (a) whole spectra and (b) magnified spectra at the RBM mode. Excitation wavelength was 532 nm.

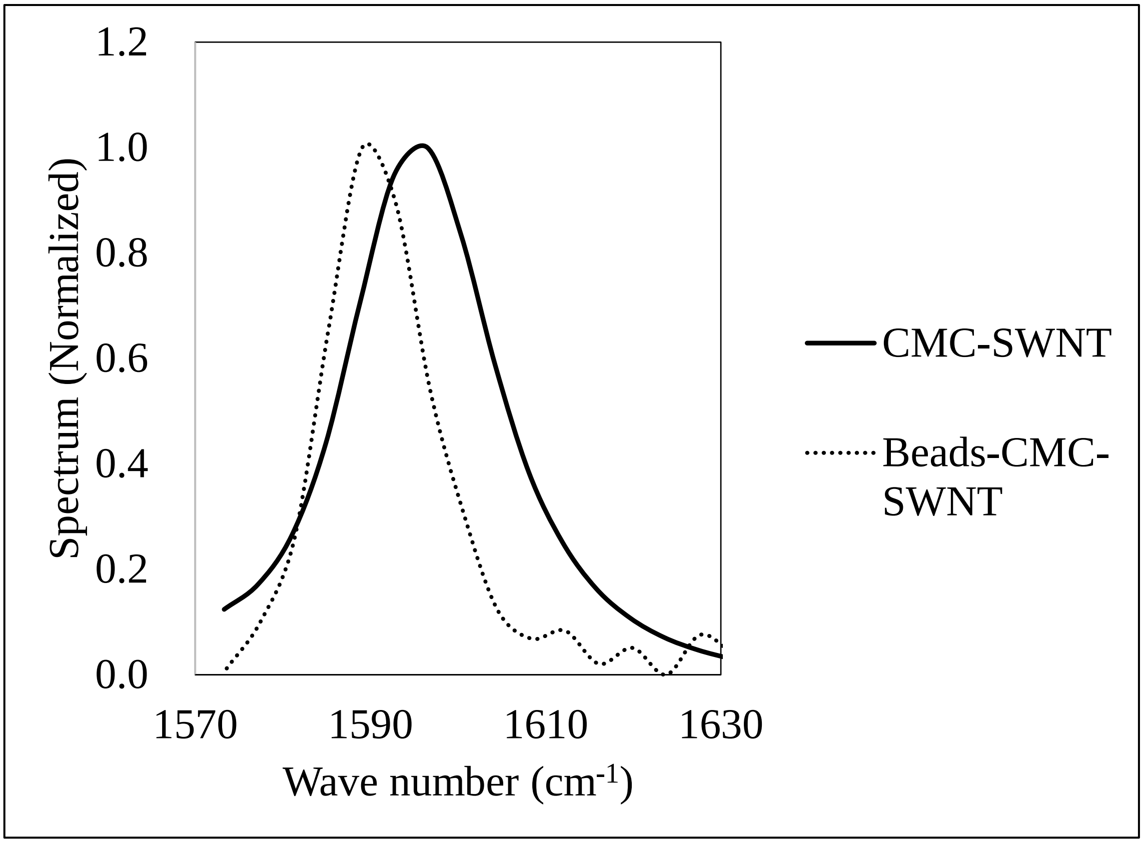

Figure S5

Peak shift of Raman spectra of CMC-SWNT. (a) CMC-SWNT. (b) CMC-SWNT attached on microbead surfaces.

Additives	Initial	Add $KMnO_4$	Add papain/PBS
(1) Un-heated papain	1	0.18 ± 0.12	0.61 ± 0.09
(2) Pre-heated papain (60 °C)	1	0.26 ± 0.21	0.60 ± 0.31
(3) Pre-heated papain (100 °C)	1	0.12 ± 0.10	0.71 ± 0.14
(4) Un-heated PBS	1	0.20 ± 0.10	0.15 ± 0.10
(5) Pre-heated PBS (100 °C)	1	0.10 ± 0.07	0.10 ± 0.10

n = 15 (papain), *n* = 9 (PBS)

Table S1
A numerical analysis of the PL intensity of microbeads functionalized with CMC-SWNT hybrids containing $KMnO_4$ and papain solutions was performed (a summary of the data is presented in Figure 3). The PL intensities of the initial states were normalized to 1.

		Average	SD	Variance	*p* value
(1)	$KMnO_4$	0.175	0.112	0.014	1.50×10^{-08}
	Papain (RT)	0.609	0.086	0.008	
(2)	$KMnO_4$	0.262	0.201	0.043	1.76×10^{-06}
	Papain (60°C)	0.596	0.298	0.095	
(3)	$KMnO_4$	0.124	0.093	0.009	7.6×10^{-10}
	Papai(100°C)	0.713	0.131	0.018	
(4)	$KMnO_4$	0.197	0.090	0.009	0.247
	PB(RT)	0.154	0.099	0.011	
(5)	$KMnO_4$	0.099	0.062	0.004	0.416
	PB(100°C)	0.105	0.097	0.011	

※RT : Room Temperature
※SD : Standard Deviation

Table S2
t-test of PL data of Fig. 3 and Table S1

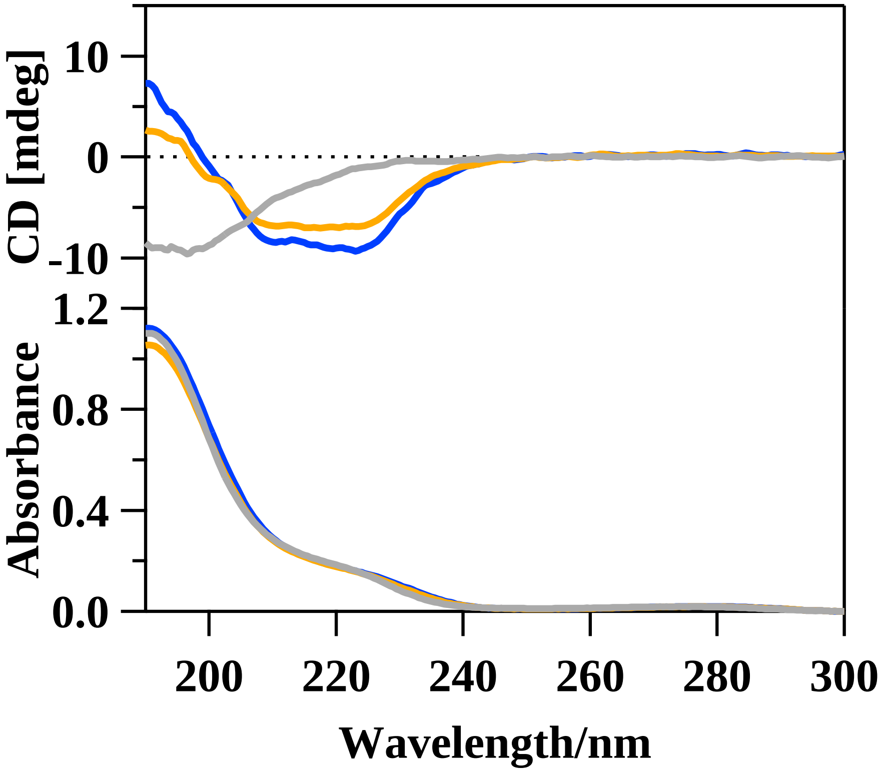

Figure S6

CD and absorbance spectra of un-heated papain (blue), papain pre-heated at 60 °C (orange), and papain pre-heated at 100 °C (gray). Scan speed: 50 nm/min. Accumulation: 4 times with smoothing.

	Un-heated papain	Pre-heated papain (60 °C)	Pre-heated papain (100 °C)
Helix	24.9	19.4	3.1
Beta	16.6	20.3	24.0
Turn	18.9	18.8	14.4
Random	39.6	41.5	58.5
RMSD	0.064	0.052	0.038

Percentage of the secondary structure motifs of papain estimated by the CONTIN/LL (48 reference set: No7) program.

Table S3

Secondary structures of papain molecules

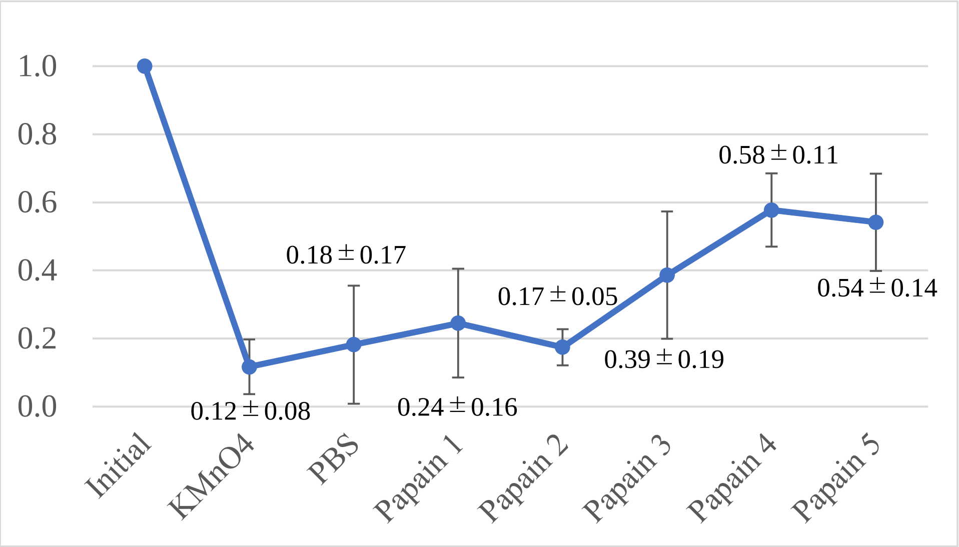

Figure S7

Sequential measurements of PL intensity of micro-beads functionalized with CMC-SWNT hybrids with $KMnO_4$, PBS, and pre-heated papain (60 °C). Summary of the data of Figure 4.
